# The BAF complex in development and disease

**DOI:** 10.1186/s13072-019-0264-y

**Published:** 2019-03-21

**Authors:** Amelie Alfert, Natalia Moreno, Kornelius Kerl

**Affiliations:** Department of Paediatric Haematology and Oncology, University Children’s Hospital Muenster, Domagkstraße 24, 48149 Muenster, Germany

**Keywords:** Rhabdoid tumour, Synovial sarcoma, Mammalian SWI/SNF complex, BAF complex, Cancer, Epigenetic, Chromatin remodelling, Neurodevelopmental disorders

## Abstract

The ATP-dependent chromatin remodelling complex BAF (= mammalian SWI/SNF complex) is crucial for the regulation of gene expression and differentiation. In the course of evolution from yeast to mammals, the BAF complex evolved an immense complexity with a high number of subunits encoded by gene families. In this way, tissue-specific BAF function and regulation of development begin with the combinatorial assembly of distinct BAF complexes such as esBAF, npBAF and nBAF. Furthermore, whole-genome sequencing reveals the tremendous role BAF complex mutations have in both neurodevelopmental disorders and human malignancies. Therefore, gaining a more elaborate insight into how BAF complex assembly influences its function and which role distinct subunits play, will hopefully give rise to a better understanding of disease pathogenesis and ultimately to new treatments for many human diseases.

## Background

Packing a DNA strand, with the length of over 1 m, into a 5-μm nucleus is obviously a challenge to be met in the course of evolution. It is known that this can only be achieved by condensation of DNA with the help of histones [1, 2]. Further compaction is engineered by additional proteins [3]. However, the DNA still needs to be accessible to the transcription machinery. In this way, organisation of DNA in chromatin is both an obstacle and an additional way of gene regulation (see Ref. [4] for a review).

Epigenetic mechanisms allow regulation of DNA expression and chromatin accessibility and include DNA methylation (see Ref. [5, 6] for reviews), histone modifications (see Ref. [7, 8] for reviews) and ATP-dependent chromatin remodelling. This review will focus on the SWI/SNF family of ATP-dependent chromatin remodelling complexes and its role in development and disease.

## Evolutionary aspects of ATP-dependent chromatin remodelling by SWI/SNF complexes

The first subunits of the ATP-dependent SWI/SNF complex were discovered in yeast, where some of its subunits were detected in two independent genetic screenings, one for genes being responsible for the regulation of mating-type switching [9] and the other for those being able to allow changing of nutrient sources used for energy supply [10–12]. With respect to these discoveries, the term SWI/SNF complex (short for SWItch/sucrose non-fermentable) was coined and is used in other species as well [13–18].

The yeast SWI/SNF complex has a molecular weight of just 1.14 MDa [19] and is composed of six core subunits (Swi2/Snf2 as ATPase subunit, Swi1, Swi3, Snf5, Snf4 and Snf6) and additional Swp- and actin-related proteins (Swp29, Swp59, Swp61, Swp73 and Swp82). Consequently, it is less elaborate than its mammalian counterpart [13, 19, 20].

In Drosophila melanogaster, Brahma (Brm) is the equivalent of the yeast Swi2/Snf2 ATPase subunit and the complex it forms with additional proteins is named BAP (short for Brahma-associated protein) complex [21–23]. This complex was first discovered in screens to uncover genes that are able to suppress phenotypes caused by mutations in Polycomb genes [23, 24].

Mirroring the increasing complexity of the mammalian genome compared to yeast and fly, the mammalian equivalents of the earlier discovered ATP-dependent chromatin remodellers also became more diverse [25]. The BAF complex is one of four ATP-dependent chromatin remodelling complex families known in mammals (the others being INO80/SWR1, ISWI and CHD) (see Ref. [26] for a review). In the course of evolution, some of the yeast SWI/SNF subunits stayed conserved in mammalian genes encoding the BAF complex subunits BAF250a/b (homologues of Swi1), BRG1/BRM (Swi2), BAF155/170 (Swi3), BAF60 (Swp73), BAF53 (ARP7/9), BAF47 (Snf5) and the BAF45 family (Swp 82). More recently evolved subunits such as BCL7a/b/c, BCL11a/b, BRD7/9 and SS18/CREST lack homologues in yeast [18, 27, 28].

Taking into account that the 2 MDa mammalian BAF (short for BRG1/BRM-associated factor) complex contains up to 15 subunits [18, 25, 27, 29] and that many of these subunits are encoded by gene families and can therefore be replaced by their paralogues, it becomes clear that there are hundreds of potential assemblies possible for mammalian BAF complexes. At the same time, the high stability of subunit binding amongst each other prevents frequent subunit alterations [27, 30]. Thus, the combinatorial assembly becomes a way to ensure complex specificity and allows the BAF complex to perform the more elaborate regulation the mammalian genome requires. Many of the resulting complexes are unique to specific tissues or biological functions such as neural development and function [31–34], heart development [35, 36], muscle development [37–39] or embryonic stem cell pluripotency [40, 41]. Hence, it is not only the BAF complex itself that controls biological processes but the expression of distinct BAF complexes with unique subunit compositions is also a major part of the regulatory process.

BRG1 or BRM is incorporated into the complex as catalytic subunits with ATPase activity [18, 25], and in particular, their helicase domains show a high grade of conservation [42, 43]. Furthermore, BRG1 or BRM alone is able to remodel nucleosome templates in vitro without being accompanied by other subunits. Adding three other dedicated members of the BAF complex, namely BAF47, BAF155 and BAF170, this core complex reaches a remodelling activity that resembles the activity of the entire SWI/SNF complex [44]. Nonetheless, Mashtalir et al. recently questioned the idea of a core complex consisting of the ATPase subunit, BAF47, BAF155 and BAF170. Their studies revealed that the assembly of the BAF complex begins with the dimerisation of a BAF155::155 homodimer or a BAF155::170 heterodimer. This is the platform for further BAF assembly, and the core module is formed by first incorporating BAF60 and later BAF47 and BAF57. The next steps are the integration of BAF250 and later BAF45C. Not until this core intermediate is formed, can the ATPase module (consistent of BRG1 or BRM, actin, SS18, BRD7 and BAF53A) bind and complete the BAF complex [45]. Both core subunits and variable ones contain DNA- and/or histone-binding domains such as zinc fingers, AT-hooks and chromo- and bromodomains. As a result, BAF complexes do not only recognise binding sites based on DNA sequence but more importantly also based on architectural characteristics and pre-existing regulatory histone modifications [18, 46, 47] (see [48] for a review).

Concurrent with changes in subunit composition and gain in complexity in the course of evolution, the functions to be fulfilled by SWI/SNF complexes also expanded. The yeast genome mainly consists of actively expressed genes, with SWI/SNF being responsible for transcriptional activation but not for repression. In this organism, SWI/SNF predominantly targets histones and nucleosomes [16, 49, 50]. In Drosophila, on the other hand, the BAP complex mostly executes its function by opposing the Polycomb gene family [23, 51, 52]. In comparison with yeast and fly, the total number of genes encoded by the mammalian genome is only changed to a minor degree, but the amount of regulatory elements increased substantially and large parts of the genome are in a repressed state. Reflecting this circumstance, the mammalian BAF complex is able to activate and repress genes, causing a limited comparability between the yeast SWI/SNF complex and those in more complex multicellular organisms [53–55].

Based on different subunit composition, two distinct BAF complexes have already been described. The PBAF (Polybromo-associated BAF complex) can be distinguished from the cBAF (canonical BAF complex) by the incorporation of BAF200 instead of BAF250A/B and of BAF180 [18]. Furthermore, PBAF lacks SS18 but includes the PBAF-specific subunits BAF45A and BRD7 [54, 56]. Nonetheless, most recent studies question the existence of only two distinct BAF complex subgroups by describing a third class, called ncBAF (for non-canonical BAF complex) or GBAF (after its distinctive subunits GLTSCR1/1L) [57]. It lacks many of the dedicated cBAF subunits such as BAF47, BAF57 and BAF250 and the PBAF-specific subunits BAF180 and BRD7. In addition, it always contains a BAF155::155 homodimer and BAF60A instead of BAF60B or 60C and is further characterised by the incorporation of BRD9 and GLTSCR1/1L [45, 57, 58].

## The role of BAF complexes during mammalian development

As already mentioned, distinct subunit compositions occur at different time points during development and in different tissues, underlining the importance of combinatorial assembly in functional specificity of the BAF complex. Three especially well-studied complex assemblies are esBAF, npBAF and nBAF.

### esBAF complex and its role in embryonic development

Embryonic stem cells (ESCs) are characterised by the ability to self-renew and to differentiate into all cell lineages of the adult organism. This is, in part, achieved by the expression of pluripotency-related transcription factors such as OCT4, SOX2 and NANOG [59–63]. Since the genetic code remains unaltered in all tissues, the relevance of epigenetic control of chromatin assembly and accessibility as well as of gene expression itself becomes exceedingly clear. Besides these unique abilities of embryonic stem cells, they are also characterised by a unique chromatin structure (e.g. a high amount of bivalent domains [64]). The assembly of an ES cell-specific BAF (esBAF) complex is required for regulation of the ES cell transcriptome. The esBAF complex is, in contrast to BAF complexes in other cells, marked by the dependency on BRG1 as ATPase subunit (while BRM is not included in the esBAF complex). Moreover, it is distinguishable by the incorporation of Baf250a not 250b, Baf60a/b not 60c and a Baf155::155 homodimer instead of a Baf155::170 heterodimer in murine ESC [41, 54]. In human ESC BAF170, and not BAF155, seems to play an important role in the maintenance of pluripotency [65].

A possible way of elucidating the role and importance of individual subunits of multiprotein complexes in vivo is the creation of mouse strains that lack these subunits. Unfortunately, loss of Brg1, Baf155 or Baf47 is lethal to these animals at a very early embryonic stage. Both *Brg1* and *BAF155* knockouts are peri-implantationally lethal and *Baf47*-depleted embryos do not survive beyond day 7.5 [66–69]. Even if this fact emphasises the importance of these subunits for early embryonic development and stem cell function, it makes further research particularly difficult. Interestingly, *Brm*-knockout mice reach adulthood and are fertile with the only difference to their littermates being a slightly increased body size [70].

Regardless of the lethality of core-subunit loss in vivo, (conditional) knockdown (KD) or knockout (KO) experiments of distinct esBAF subunits proved themselves to be a promising technique to study their role in in vitro experiments with embryonic stem cells. Depleting cells of Brg1 leads to both a loss of self-renewal and a decrease in proliferation followed by a diminished expression of the core pluripotency-related factors Oct4, Sox2 and Nanog and loss of pluripotency. A *Baf155* knockout in ESCs results in a similar phenotype [41]. Corresponding to the unique subunit composition of esBAF, neither Brm nor Baf170 overexpression can rescue *Brg1* or *Baf155* knockout, respectively. A forced expression of Baf170-containing complexes also results in the inability of the transfected cells to form teratoma, indicating a loss of pluripotency [41, 67]. This cannot be observed when depleting mouse embryonic fibroblasts (MEFs) or glial cells of *Brg1* [31, 67], once again highlighting the distinct requirements of the ES cell genome.

Furthermore, chromatin immunoprecipitation experiments followed by sequencing (ChIPseq) allowed a more specific detection of esBAF binding to the genome. It was detected that Brg1 binds to approximately four percentage of the mouse genome with binding sites located in genic and promoter regions as well as in intergenic regions. Both Brg1 and Baf155 are enriched near the transcriptional starting site (TSS) and at least in part resemble the binding patterns of core pluripotency factors Oct4, Nanog and Sox2 [40]. Murine ESC pluripotency is also ensured by the leukaemia inhibitory factor (Lif) and the Stat3 pathway that is activated by Lif. STAT3 signalling also plays a role in human embryonic stem cell pluripotency [71–73]. Interestingly, Brg1- and Stat3-binding sites display a substantial genome-wide overlap in mESC. Changes in gene transcription upon *Brg1* knockout resemble those being caused by LIF withdrawal. Stat3 binding is considerably impaired in *Brg1*-depleted ES cells. This causes loss of Stat3 binding at over 80% of sites bound by Stat3 in wild-type cells [53]. In the same publication, Ho et al. also observed changes in Polycomb function that followed Brg1 loss and will be discussed in the following.

In addition to the long-known esBAF, the newly discovered ncBAF complex also plays an important role in the regulation of the ESC transcriptome. Gatchalian et al. showed that ncBAF and esBAF differ in their localisation—while esBAF preferably binds to H3K4-monomethylated enhancers as well as to super enhancers, ncBAF seems to prefer H3K4-trimethylated promoter regions. One of the most striking differences is, however, that ncBAF binds to TAD (topologically associating domain) boundaries and CTCF sites and might in this way play a role in the regulation of chromatin organisation [58, 74].

As already mentioned, esBAF functions in close association with the core pluripotency factors Oct4, Nanog and Sox2 [40]. ncBAF, on the contrary, seems to function by distinct mechanisms. It is associated with the transcription factors Klf4 (Kruppel-like factor 4) as well as with Sp5 (specificity protein 5). This interaction allows the ncBAF complex to protect naïve pluripotency and to prevent ESC priming towards epiblast ESCs [58]. In the same publication, Gatchalian et al. also hypothesised that Brd9 is essential for chromatin binding and might, in association with Brd4, replace BAF47 in its function to guide the ncBAF complex to its target genes.

Nonetheless, the ncBAF complex is not only restricted to ESCs but can also be found in other cell lines like HEK293T as well as in synovial sarcoma and malignant rhabdoid tumour cell lines [74].

### npBAF and nBAF complexes in neural development

Neural development is a well-studied example of how specification of BAF complexes is achieved by distinct combinatorial assembly. During differentiation from ESCs to neural stem cells (NSCs), the neural progenitor BAF (npBAF) complex evolves and, accompanied by the final neurogenic cell division, changes into the neuronal BAF (nBAF) complex. Compared to the BAF subunits in ES cells, some subunits (Baf45a/d, Baf53a and SS18) are preserved and ensure self-renewal and proliferation of the cells that give rise to the nervous system, while others undergo changes. Brm can be incorporated as ATPase subunit instead of Brg1 and so can Baf250a be replaced by 250b. Instead of the Baf155::155 homodimer, the npBAF complex can include a Baf155::170 heterodimer and Baf60c replaces BAF60a/b [31, 34, 75].

Heterozygous loss of *Brg1* and *Baf155* leads to defects in neural tube closure [67, 76]. Furthermore, *Brg1* depletion in Nestin^+^–NSCs results in severe defects of proliferation and formation of the neural progenitor pool, causing thinning of the cortex and midbrain as well as a deficiency in cerebellar development, leading to perinatal death of the animals [77, 78]. Consistent with findings in ESCs, loss of subunits specific to npBAF (Baf53a, Baf45a/d and SS18) gives rise to proliferation defects in NCSs. Whereas ES cells are still able to proliferate, when being depleted of *SS18* until only 20% of wild-type levels are left, NSCs are much more sensitive to *SS18* knockdown, losing their ability to self-renew after a reduction of 25% compared to wild-type levels [75].

The shift from npBAF to nBAF coincides with the mitotic exit of neural precursors and is distinguishable by the replacement of Baf53a by 53b, SS18 by CREST, Baf45a/d by Baf45b/c and changed expression levels of Baf155 and Baf170 [31, 32, 79]. This switch has to be regulated strictly, as both premature and delayed expression of nBAF-specific subunits cause severe phenotype alterations, either showing a decrease in proliferation (see above) or disturbances in dendritic processes [80]. This is, most likely, achieved by a regulatory circuitry involving neural-specific miRNAs. miRNA9* and miRNA124 are both specifically expressed in neural cells, and their expression is repressed by REST (repressor element-1-silencing transcription factor) in neural progenitors [81–84]. Even if both these miRNAs can, in theory, target the 3′-UTR of BAF53a, they do not influence its expression as long as REST inhibits their action [85]. In postmitotic neural cells, however, REST itself is repressed by RAR (unliganded retinoic acid receptor complex) [86]. In this way, miRNA9* and miRNA124 can target BAF53a, which leads to its degradation and loss from the npBAF complex. Additionally, BAF53a expression seems to be linked directly to BAF53b repression, and following the loss of BAF53a, BAF53b is expressed [85]. This regulatory mechanism was shown to be so powerful that it is possible to convert fibroblasts into functional neurons by overexpressing miRNA9* and miRNA124 in these cells [75, 87]. For a more detailed description of the role of miRNA9* and miRNA124, see Ref. [88].

nBAF subunits have an enormous influence on various aspects of neural development and plasticity. They are essential for dendritic morphogenesis [79, 89]. Bcl11b regulates neuronal subtype maturation [90, 91], and Baf53b is involved in learning and long-term memory [33]. Moreover, BAF complexes in general control adult neurogenesis [92, 93], gliogenesis [92, 94] and neural morphogenesis [34, 79, 95–97].

**Fig. 1:**
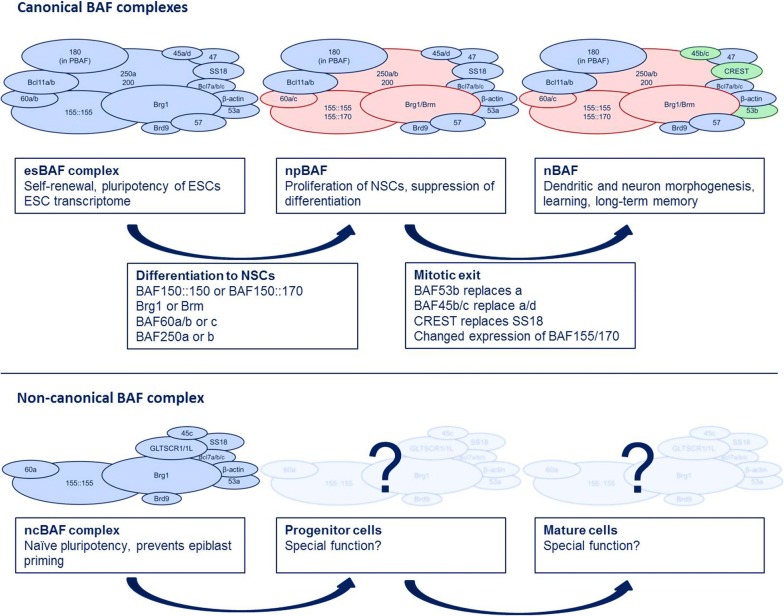
BAF complex subunit switches during mammalian development. BAF complex subunits undergo distinct switches during development to adapt to the requirements of more differentiated cell types. While the esBAF complex can only be found in embryonic stem cells and incorporates BRG1 as ATPase subunit, npBAF complexes can be found in neural progenitor cells and nBAF complexes first occur with mitotic exit. Colours are used to indicate the changes in subunit compositions. Most strikingly, npBAF can include BRM as alternative to BRG1, BAF250b as alternative to a, BAF60c as alternative to c and a BAF155::170 heterodimer to replace the BAF155::155 homodimer. nBAF-specific subunits are BAF53a, BAF45b/c and CREST. The ncBAF coexists with the esBAF complex in ESCs and has been shown to regulate naïve pluripotency by interacting with the transcription factors KLF4 and Sp5. It is characterised by the lack of many esBAF-specific subunits such as BAF250a, BAF47 and BAF57 and the incorporation of BRD9 and the ncBAF-specific subunit GLTSR1/L1. Until now, ncBAF complexes, apart from ESCs, could also be found in rhabdoid and synovial sarcoma tumour cell lines as well as in HEK293T cells

Nonetheless, not only embryonic stem cells and neural development are dependent on BAF complex regulation. Baf60c-containing complexes, for instance, are essential for heart development, and they mark embryonic tissues with cardiogenic potential and, in association with tissue-specific factors, administer the differentiation from fibroblasts to cardiomyocytes [35, 36, 98]. Similar principles are valid for skeletal muscle development [99], and it is probable there are many more specific BAF complexes yet to be discovered. In Fig. 1, an overview of BAF subunit switches during mammalian development is shown.

## Mechanistic insights into chromatin remodelling by BAF complexes

From yeast ATP remodelling complexes, it is known that they primarily fulfil their function by mobilising and exchanging nucleosomes or by moving them across the DNA [100]. On account of the structural similarities between many yeast and mammalian subunits, it was presumed that this is also true for mammalian BAF complexes. One of the first hypotheses concerning the mechanistic way ATP-dependent chromatin remodelling works was the “twist diffusion” model that postulates that the nucleosomes slide alongside the DNA strand as a reaction to the DNA twisting around the nucleosome. However, it was shown that experimental setups, that should be large obstacles for this twisting (such as biotin crosslinks, DNA hairpins or nucleosomes linked to magnetic beads), failed to inhibit nucleosome remodelling in vitro [101, 102]. Following these experiments, the “loop recapture” model was developed. It assumes that the DNA forms a loop via the formation of new contacts to histones and in association with the translocase activity that many ATPase subunits (also from other remodelling complexes) have, loop formation allows a shift of nucleosomes alongside the DNA [102–106].

Regardless of these remodelling mechanisms, it is remarkable that most of the BAF subunits are not needed for in vitro chromatin remodelling [44]. Nonetheless, loss of subunits not needed for in vitro function, such as Baf53a or Baf250a, causes severely altered phenotypes resembling those of Brg1 loss, while there are many amongst the most frequently mutated subunits in cancer that are not necessary for in vitro function [107–109]. This clearly indicates that the exact mechanisms of chromatin remodelling are not yet understood and that there is also the need for a better model to study chromatin remodelling in vitro.

### BAF–Polycomb opposition as an important mechanism of chromatin remodelling

The Polycomb gene family has first been discovered in Drosophila followed by the observation of male flies with ectopic sex combs [110–115].

In mammals, the multiprotein-containing Polycomb repressive complexes (PRC) have repressive influence on the genome. Similar to the BAF complexes in mammals, there are several different subunit compositions possible. PRC1 and PRC2 work in a rather different manner. PRC1 is (in its canonical form) composed of CBX, PHC, PCGF, RING and SCMH proteins with each having various variants. Additional diversity is enabled by the assembly of non-canonical complexes, in which SCMH is replaced by RYBP or YAF2. It typically represses gene expression by transferring a single ubiquitin to lysine 119 of histone 2A (H2AK119ub1) (see [28] for a review). PRC2 is formed by at least five subunits, namely EZH1 or 2 as catalytic subunit, EED, SUZ12, RBBP4/7 and AEBP2, with EZH1/2, EED and SUZ12 being indispensable for complex function [116, 117]. EZH1 and 2 both catalyse the SAM-dependent trimethylation of lysine 27 of histone 3. They are the only known mammalian enzymes capable of depositing this repression mark [117–119].

In Drosophila, it is well known that BAP complexes mainly function by opposing Polycomb genes [23, 24]. In recent years, it also became evident that the same principle can be found in mammalian cells as well and that this opposition is important for normal cell function. Its disruption can be responsible for tumour formation (see below). In embryonic stem cells, loss of Brg1 leaves overall expression levels of PRC2 subunits and genome-wide H3K27me3 levels unchanged. Nonetheless, there are substantial changes following knockdown: Brg1-repressed genes show a significant decrease in H3K27me3, whereas Brg1-activated genes show elevated levels of this repressive histone mark. It is shown that Polycomb and BAF complexes oppose each other at nearly all gene loci except for the Hox loci, where both act synergistically in mESCs. The phenotype caused by *Brg1* deletion can be rescued by knockdown of *Suz12*, indicating the important role the loss of Polycomb opposition has for the changes of chromatin accessibility [53].

In order to be able to study chromatin remodelling processes in a more precise and a more realistic manner, a new method has been developed that enables experiments in mouse cells instead of using artificial nucleosome templates. This model is called CiAO (short for chromatin in vivo assay at *Oct4*) and allows the recruitment of the BAF complex to the *Oct4* locus by a chemical inducer of proximity (like rapamycin), followed by the investigation of the effects of this recruitment [120]. Utilising this technique, it is shown that minutes after recruitment of BAF to *Oct4*, PRC2 is removed, with its accompanying mark H3K27me3 disappearing about 10 min after PRC2 loss. PRC1 eviction from the *Oct4* locus can be detected even earlier and coincides with parallel loss of H2AK119ub1. The corollary is also true, in that BAF removal is rapidly followed by Ezh2 (PRC2) and H3K27me3 reappearance [121]. This “indirect” mechanism of chromatin remodelling is dependent on Brg1 and its ability to bind PRC1 subunits directly. Loss of Brg1 causes weakening of the interaction and increases occupancy of PRC1 and PRC2. Furthermore, the opposition of Polycomb complexes is ATP dependent, illustrated by the fact that mutations in the ATPase domain cause the same epigenetic changes as a complete loss [122]. Being most relevant for the explanation of cancer formation, even a heterozygous mutation of the ATPase domain is sufficient to significantly alter chromatin accessibility [123]. The consequences of other cancer-related changes in BAF–Polycomb opposition will be discussed in the following.

## The role of BAF complexes in human disease

### The BAF complex in neurodevelopmental disorders

With regard to the importance of the BAF complex and distinct subunit switches in neural development, it is not surprising that mutations in BAF-coding genes are associated with neurodevelopmental disorders such as Coffin–Siris syndrome, Nicolaides–Baraitser syndrome or autism spectrum disorders.

One of the syndromes commonly caused by mutations in BAF subunits is the Coffin–Siris syndrome (CSS), first described in 1970 as a combination of intellectual disability, growth retardation, joint malformations and brachydactyly combined with hypoplastic or missing fingernails at the fifth finger/toe [124, 125]. These characteristics can be accompanied by coarse facial features, numerous organ abnormalities (cardiovascular, gastrointestinal or genitourinary) and feeding difficulties [126, 127]. The most frequently mutated gene in Coffin–Siris syndrome is BAF250b, being mutated (dependent on the cohort) in at least 68% of cases [128, 129]. BAF250b is also the subunit most frequently mutated in SWI/SNF-related intellectual disability disorders in general [130]. Whereas its paralogue BAF250a can also be detected in CSS [131, 132], it has a much more dominant role in cancer (see below). ATPase subunits BRM and BRG1 are also related to CSS [129, 133, 134]. BAF47, the subunit known to be responsible for rhabdoid tumours [135], gives rise to a severe form of CSS [136].

The Nicolaides–Baraitser syndrome (NCBRS) is an intellectual disability disorder less variable than CSS. It is characterised by seizures, prominent interphalangeal joints without signs of inflammation, severe intellectual disability with speech delay, growth retardation and characteristic facial features [137]. It is primarily caused by missense mutations in the alternative ATPase subunit BRM [138–140]. In conformity with the previously mentioned difference between BAF250a (mostly in cancer) and BAF250b (in neurodevelopmental disorders), BRM is the ATPase subunit predominantly associated with intellectual disabilities, while its paralogue BRG1 is more closely related to cancer (see below).

**Fig. 2:**
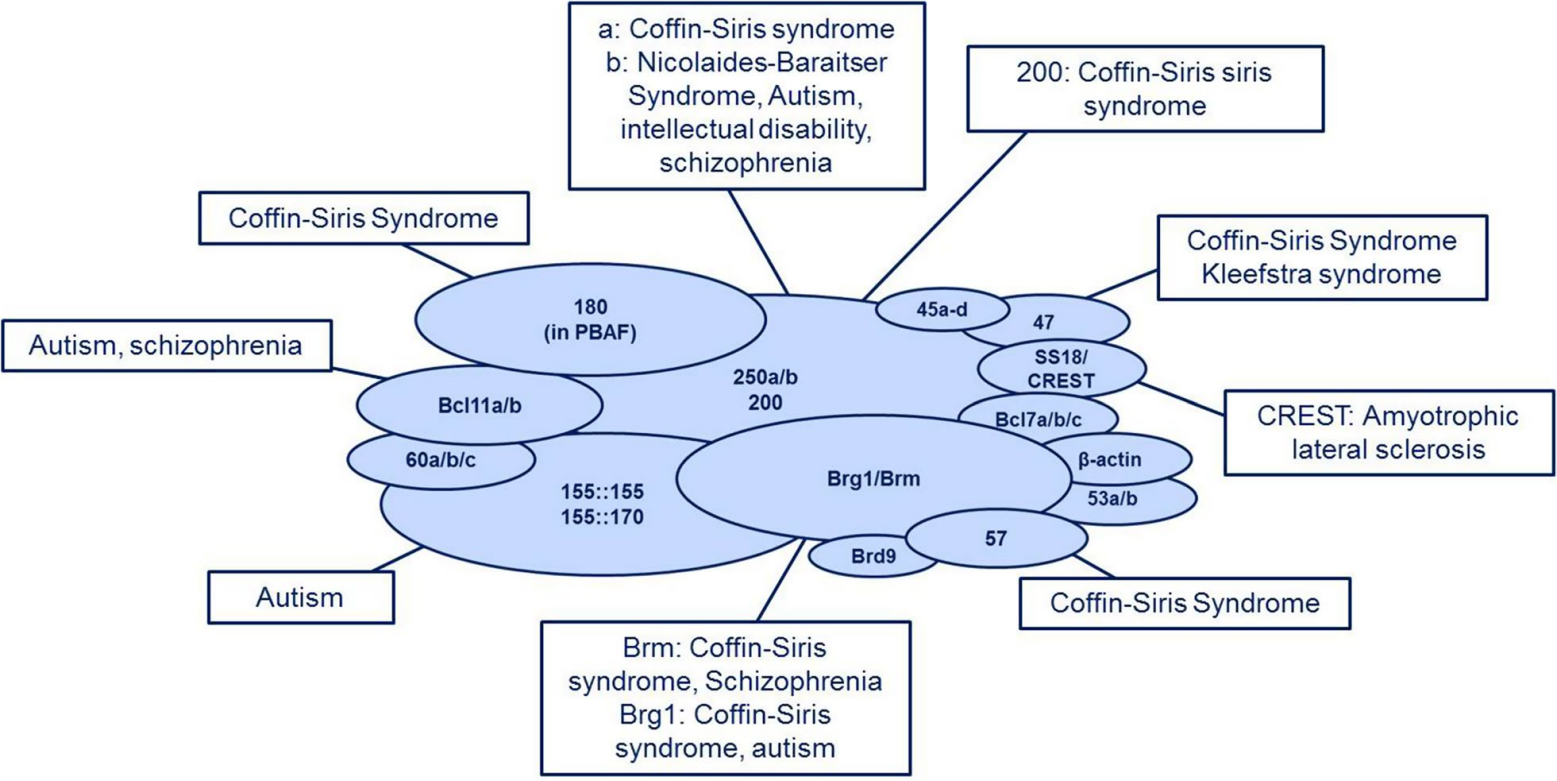
The role of subunit mutations in developmental disorders. BAF subunit mutations have a high implication in human developmental disorders. The most frequent mutations and associations with human disease are summarised in this figure. Subunits being involved most frequently include the ATPase subunit BRM as well as the subunit BAF250b

In addition to Coffin–Siris syndrome and Nicolaides–Baraitser syndrome, there are many other neurodevelopmental disorders related to BAF subunit mutations, such as Kleefstra’s syndrome [141, 142], disorders of the autism spectrum [143, 144], amyotrophic lateral sclerosis [145] and schizophrenia [146, 147]. For a more detailed review concerning the role of BAF complexes in neural developmental disorders, see Ref. [148–150]. BAF subunit mutations and their implication in human developmental disorders are summarised in Fig. 2.

### The BAF complex in cancer

Given its immense influence on both differentiation and gene expression in general, it is not surprising that BAF complex subunits are frequently altered in cancer with up to 20% of human cancers bearing mutations in this ATP remodelling complex. Thus, it is the chromatin remodelling complex most frequently involved in human malignancies [27]. Mirroring the tissue specificity of BAF complex assembly, the influence of subunit mutations depends on the BAF complex assembly and the tissue in which they occur. Some are only occasionally associated with cancer, while others show a much higher mutation frequency. Furthermore, each is related to only a distinct subset of cancer entities. Moreover, some mutations only cause tumorigenesis when biallelic loss occurs, whereas in other cases a mutation in one allele is detected [135, 151]. The best studied examples of how mutations of chromatin remodellers can cause malignancies are BAF47 (= SmarcB1) loss in rhabdoid tumours and the SS18-SSX fusion protein in synovial sarcoma. These two examples show that the BAF complex can act as both oncogene and tumour suppressor.

### The BAF complex as tumour suppressor

Rhabdoid tumours are aggressive malignancies occurring in early childhood arising in the brain (atypical teratoid/rhabdoid tumours = AT/RT), soft tissue (malignant rhabdoid tumours = MRT) or kidneys (rhabdoid tumours of the kidney = RTK). Almost all of them show a biallelic loss of *BAF47*, but have an otherwise low mutational burden with no additional recurrent mutations detectable. Thus, rhabdoid tumours can mainly be considered as an epigenetic disease caused by the loss of the recessive tumour suppressor BAF47 [135, 152]. Moreover, this clear correlation between loss of BAF47 and tumour formation can be remodelled in mice. Conditional knockout of *BAF47* at a distinct time point leads to tumour formation after 11 weeks with a penetrance of 100%, illustrating that loss of BAF47 is sufficient to drive tumour formation. Other studies have shown that loss of heterozygosity can also cause peripheral T cell lymphoma [153, 154]. In addition, BAF47 loss also occurs in a high subset of epithelioid sarcoma [155].

Mechanistically, it is known that BAF47 plays a crucial role in BAF-mediated chromatin remodelling. Upon loss of BAF47, the BAF complex itself is still able to assemble [156, 157] but displays a diminished chromatin affinity. Dissociation of BAF complex and chromatin requires harsher conditions after BAF47 rescue. Consistent with this, genome-wide BAF complex occupancy increases significantly after BAF47 rescue. Furthermore, it results in a widespread enhancer and super-enhancer activation as well as an alteration in bivalent promoter regulation [158]. As already mentioned, a key factor in BAF function is Polycomb opposition. In support of this, EZH2 has been shown to be a key player in many malignancies, including rhabdoid tumours [159] (also see Ref. [160] for a review). BAF complexes deficient of BAF47 can still be recruited to the genome but are unable to evict Polycomb, as they usually do [121]. As a result, tumour cells show an increase in the H3K27me3 repressive mark, amongst others, at the p16Ink4a tumour suppressor locus, which is known to drive rhabdoid tumour formation [159, 161]. Relevant to translational research, it has also been shown that additional inactivation of EZH2 in BAF47-deficient MEFs results in wild-type levels of p16Ink4a [159] and that tumour regression in MRTs can be achieved by chemical inhibition of EZH2 [162, 163]. Currently, a number of EZH2 inhibitors are tested in phase I/II clinical trials in other cancer entities [164, 165]. Interestingly, the expression of other BAF complex subunits like BRM seems to determine the sensitivity of tumours with BAF subunit mutations to EZH2 inhibition, suggesting BRM expression as a possible biomarker for therapeutic response [166]. For those tumours that are insensitive to EZH2 inhibition, BRM inhibition could be a promising alternative [167–169].

Despite the described role of the cBAF complex in rhabdoid tumours, there are also recent studies suggesting an auxiliary role of the ncBAF complex. Krämer et al. showed that BRD9 inhibition results in a decrease in cell proliferation as well as a G1 cell cycle arrest and an increase in apoptosis in several rhabdoid tumour cell lines [170]. This is also supported by CRISPR-Cas9-based screens, identifying BRD9, GLTSCR1 and BAF60A, all being subunits of the ncBAF complex, as critical for rhabdoid tumour cell line survival [74]. ChIPseq experiments using BRD9 and BRG1 antibodies suggest that many of the residual BAF complexes in rhabdoid tumours are, in fact, ncBAF complexes. They localise to enhancers that have already been identified as tumour associated and rhabdoid tumour specific. In this way, ncBAF preserves gene expression at CTCF sites as well as in promoter–proximal regions. The unique dependency of rhabdoid tumours on ncBAF complex function might make BRD9 or GLTSCR1 inhibition an additional promising target for rhabdoid tumour treatment [74].

There are also other cancer entities, in which the BAF complex loses its tumour-suppressive abilities. Mutations of BRG1, for instance, occur in over 90% of small cell ovarian cancers [171, 172]. BAF250a mutations, the subunit most frequently mutated in human malignancies, can be found in a huge subset of cancers, e.g. endometrial carcinoma, gastrointestinal carcinoma (colorectal, gastric), pancreatic carcinoma and cholangiocarcinoma [173–178]. Amongst the subunits less frequently involved in cancer are BAF170 (in gastric and colorectal cancer with microsatellite instability) [179], BAF155 (in small cell lung cancer) [27] as well as BAF45d and BAF60 in breast cancer [31, 180].

### The BAF complex as oncogene: the SS18-SSX fusion in synovial sarcoma

Similar to rhabdoid tumours, synovial sarcomas are tumours in which one single, well-characterised mutation can be found in nearly all patients. It is an aggressive sarcoma arising in the soft tissue and specified by a t(X;18) chromosomal translocation, fusing 78 amino acids of the protein SSX to the dedicated BAF complex subunit SS18 [151, 181, 182]. Nonetheless, the mechanism underlying the transformation is an entirely different one. Unlike rhabdoid tumours, which are caused by a biallelic loss of *BAF47*, tumour formation in synovial sarcoma occurs in spite of a remaining wild-type allele. This might be possible because the transcription of the wild-type allele is decreased in sarcoma cells in general. Additionally, the SS18-SSX fusion protein is preferably incorporated into the complex, leading to the degradation of the monomeric wild-type protein [27, 183]. Besides, BAF47 is almost completely lost from the BAF complex upon SS18-SSX incorporation, but in contrast to rhabdoid tumours, the ability to oppose PRC1 and PRC2 is preserved regardless of the changed subunit composition [121, 151]. Furthermore, BAF47 rescue and the following restoration of enhancer action are not necessary to decrease proliferation in synovial sarcoma [184]. Therefore, synovial sarcoma is primarily driven not by BAF47 loss, but by the gain of SS18-SSX which seems to be the driving force.

**Fig. 3:**
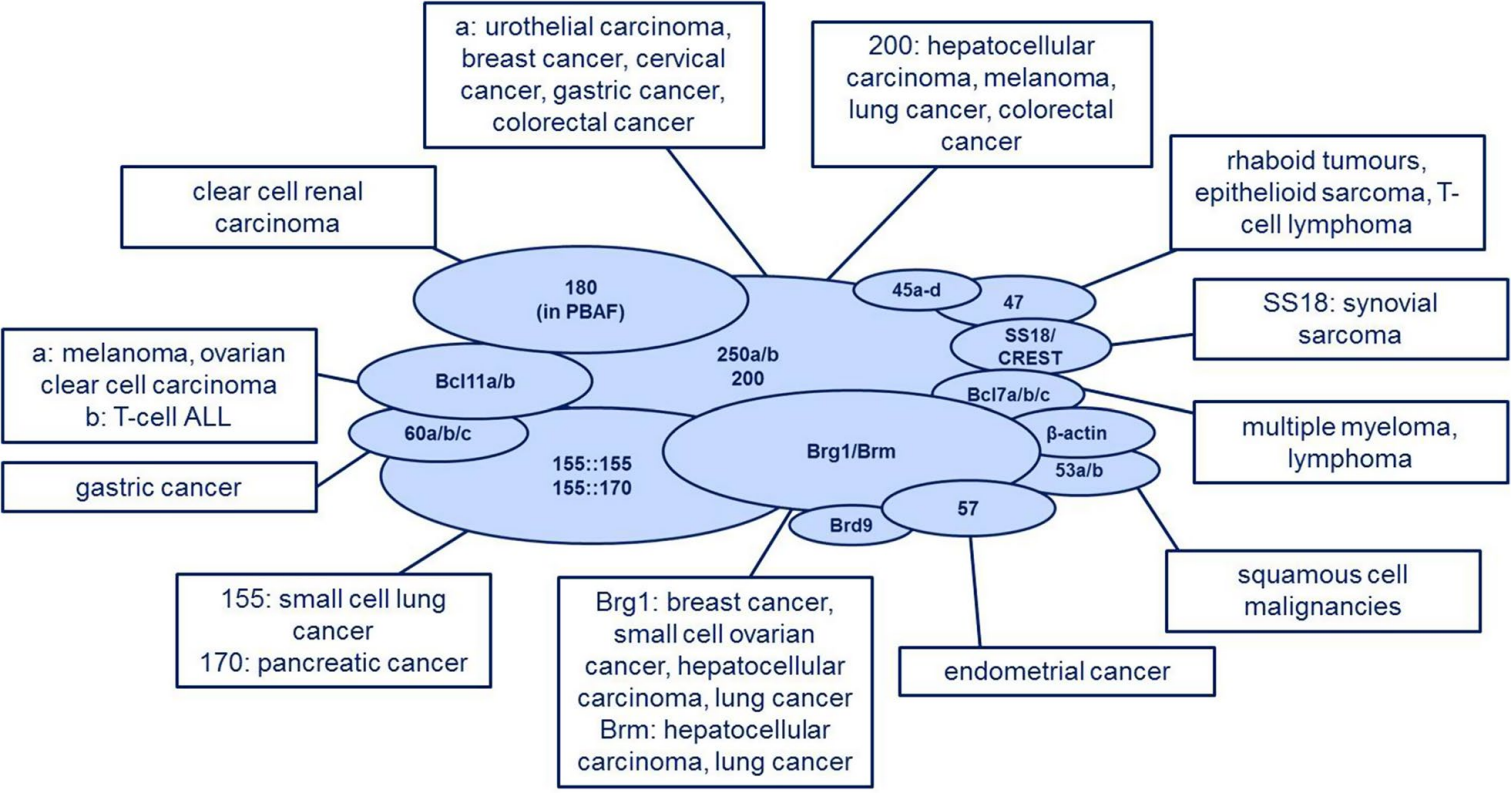
The role of subunit mutations in cancer. Mutations in BAF subunit do not only cause neurodevelopmental diseases but also have an important role in tumorigenesis. Genome-wide sequencing revealed that about 20% of human cancers show BAF subunit mutations; amongst the most frequently mutated subunits is BAF250a. Like BAF complex composition unique to some tissue types, many mutations have a “unique” pattern of malignancies they play a role in

Indeed, SS18-SSX causes broad changes in BAF complex targeting and results in loss and, perhaps more importantly, a gain of chromatin occupancy by BAF complexes. SS18-SSX-containing BAF complexes show an unusual co-occupancy with PRC2, amongst others, at the *SOX2* and *PAX3* loci, which results in the eviction of Polycomb and the loss of H3K27me3 [121, 184]. These changes are sufficient to cause SOX2 expression, which is a typical feature of synovial sarcoma cell lines, that show stem cell-like expression patterns [151, 185]. Moreover, recent studies indicate that the SS18-SSX-containing BAF complex interacts with KDM2B and uses its demethylase activity to activate genes usually repressed [186]. Regardless of the substantial changes the SS18-SSX fusion protein gives rise to, the original assembly of the BAF complex (containing SS18 and BAF47) can be rescued by either overexpression of wild-type *SS18* or *SS18*-*SSX* knockdown. Both lead to a proliferation arrest [151, 184]. Being mindful of the immense effect the specific inhibition of the fusion gene bcr-abl has in CML (chronic myelogenous leukaemia) [187, 188], targeted therapy with inhibitors against SS18-SSX could be a new approach to treat synovial sarcoma, as already suggested by Kadoch and Crabtree [189]. Figure 3 shows an outline of BAF subunit mutations in human cancers.

Similar to the findings in rhabdoid tumours, ncBAF also seems to play a significant role in synovial sarcoma as cell lines of this tumour entity are sensitive to the loss of BRD9, BAF60A or GLTSCR1. SYO-1 cells react with a decrease in proliferation, a G1 phase arrest and an increase in apoptosis upon treatment with BRD9 inhibitors. Other sarcoma cell lines do not show this dependency on BRD9 which makes it a specific target in cancers with cBAF perturbations [74, 190]. In contrast to the canonical BAF complex, ncBAF preferably incorporates SS18 not SS18-SSX [74]. Nonetheless, other experiments could show the existence of BRD9 and SS18-SSX-containing complexes with BRD9 and SS18-SSX co-binding on the genome [190]. Even if the cells are clearly dependent on ncBAF function, BRD9 inhibition neither inhibits SS18-SSX-mediated gene activation nor is it required for the de novo activation of these genes by SS18-SSX. Unlike cBAF, ncBAF is only influenced in its localisation to a minor degree. It still localises to H3K4-trimethylated regions as well as to CTCF sites and most genes that are downregulated following BRD9 inhibition are independent from the SS18-SSX fusion protein. For this reason, Michel et al. [74] suggested that ncBAF might be responsible for maintaining the gene expression of indispensable genes that are no longer activated by the re-targeted SS18-SSX-containing BAF complex. The dependency of synovial sarcoma on BRD9 could make BRD9 inhibition a promising target for therapy of this cancer entity. For this purpose, targeted BRD9 degradation might even be more effective than the inhibition of its bromodomain [190].

## Conclusion

Evidently, a functioning BAF complex is indispensable to both differentiation of embryonic stem cells into mature cell lines and regulation of the transcriptome of these cells. Even if there has been an enormous gain in understanding how BAF complexes assemble and function in vitro, their in vivo function and the role of distinct subunits still remain an enigma. Why different mutations of the same subunit, e.g. BAF47, can cause neurodevelopmental disorders or human malignancies, is still unclear. Furthermore, the question why some subunits only cause tumour growth when both alleles are lost, while in other tumours only heterozygous mutations are detected, continues to be unanswered.

However, the discovery of EZH2 inhibitors and BRD9 inhibitors as possible therapeutic approaches for some cancers with BAF subunit mutations, illustrates why the comprehension of the precise mechanism by which subunit mutations result in tumour growth, is so important. Currently, therapeutic options of many cancers are limited by the toxicity of the therapy itself and can scarcely be made more aggressive. Therefore, developing targeted therapies that address the distinct mechanisms by which tumour growth can be driven is indispensable to improving patient survival. However, these mechanisms first need to be fully understood making further elucidation of how BAF subunit mutations drive tumour formation essential. Consistent with this, the cause of neurodevelopmental disorder cannot be understood without understanding the role of BAF complexes in early and late neurogenesis.

## Abbreviations

AT/RT: atypical teratoid/rhabdoid tumours
BAF complex: BRG/BRM-associated factor complex
BAP complex: Brahma-associated protein complex
ChIP: chromatin immunoprecipitation
ChIPseq: chromatin immunoprecipitation followed by sequencing
CiAO: chromatin in vivo assay at Oct4
CML: chronic myelogenous leukaemia
CSS: Coffin–Siris syndrome
esBAF complex: embryonic stem cell-specific BAF complex
ESC: embryonic stem cell
LIF: leukaemia inhibitory factor
MEF: mouse embryonic fibroblast
MRT: malignant rhabdoid tumours
nBAF complex: neuronal BAF complex
NCBRS: Nicolaides–Baraitser syndrome
npBAF complex: neural progenitor BAF complex
PRC: Polycomb repressive complex
RTK: rhabdoid tumours of the kidney
SWI/SNF: SWItch/sucrose non-fermentable
